# Stewardship-Guided T2Candida Testing Shortens Time to Antifungal Treatment and Reduces Antifungal Usage Among Medical Intensive Care Unit Patients With Septic Shock

**DOI:** 10.1093/ofid/ofad538

**Published:** 2023-11-08

**Authors:** Matthew O’Donnell, Ryan K Shields, Rachel V Marini, Lara M Groetzinger, Brian A Potoski, Bonnie A Falcione, Sunish Shah, Erin K McCreary, Lloyd Clarke, Emily Brant, Bryan J McVerry, Susan Liegey, A William Pasculle, Cornelius J Clancy, M Hong Nguyen

**Affiliations:** Department of Medicine, University of Pittsburgh, Pittsburgh, Pennsylvania, USA; Division of Infectious Diseases, UPMC, Pittsburgh, Pennsylvania, USA; Antibiotic Management Program, UPMC, Pittsburgh, Pennsylvania, USA; Department of Critical Care Medicine, UPMC, Pittsburgh, Pennsylvania, USA; Department of Medicine, University of Pittsburgh, Pittsburgh, Pennsylvania, USA; Division of Infectious Diseases, UPMC, Pittsburgh, Pennsylvania, USA; Antibiotic Management Program, UPMC, Pittsburgh, Pennsylvania, USA; Department of Pharmacy, UPMC, Pittsburgh, Pennsylvania, USA; Division of Infectious Diseases, UPMC, Pittsburgh, Pennsylvania, USA; Antibiotic Management Program, UPMC, Pittsburgh, Pennsylvania, USA; Department of Pharmacy, UPMC, Pittsburgh, Pennsylvania, USA; Department of Pharmacy, UPMC, Pittsburgh, Pennsylvania, USA; Department of Medicine, University of Pittsburgh, Pittsburgh, Pennsylvania, USA; Division of Infectious Diseases, UPMC, Pittsburgh, Pennsylvania, USA; Antibiotic Management Program, UPMC, Pittsburgh, Pennsylvania, USA; Department of Pharmacy, UPMC, Pittsburgh, Pennsylvania, USA; Department of Pharmacy and Therapeutics, School of Pharmacy, University of Pittsburgh, Pittsburgh, Pennsylvania, USA; Department of Medicine, University of Pittsburgh, Pittsburgh, Pennsylvania, USA; Division of Infectious Diseases, UPMC, Pittsburgh, Pennsylvania, USA; Antibiotic Management Program, UPMC, Pittsburgh, Pennsylvania, USA; Department of Pharmacy, UPMC, Pittsburgh, Pennsylvania, USA; Department of Pharmacy and Therapeutics, School of Pharmacy, University of Pittsburgh, Pittsburgh, Pennsylvania, USA; Division of Infectious Diseases, UPMC, Pittsburgh, Pennsylvania, USA; Antibiotic Management Program, UPMC, Pittsburgh, Pennsylvania, USA; Department of Pharmacy, UPMC, Pittsburgh, Pennsylvania, USA; Division of Infectious Diseases, UPMC, Pittsburgh, Pennsylvania, USA; Antibiotic Management Program, UPMC, Pittsburgh, Pennsylvania, USA; Department of Critical Care Medicine, UPMC, Pittsburgh, Pennsylvania, USA; Department of Medicine, University of Pittsburgh, Pittsburgh, Pennsylvania, USA; Division of Pulmonary, Allergy, Critical Care, and Sleep Medicine, UPMC, Pittsburgh, Pennsylvania, USA; Division of Clinical Microbiology, UPMC, Pittsburgh, Pennsylvania, USA; Division of Clinical Microbiology, UPMC, Pittsburgh, Pennsylvania, USA; Department of Pathology, University of Pittsburgh, Pittsburgh, Pennsylvania, USA; Department of Medicine, University of Pittsburgh, Pittsburgh, Pennsylvania, USA; Veterans Affairs Pittsburgh Healthcare System, Department of Medicine, Division of Infectious Diseases, Pittsburgh, Pennsylvania, USA; Department of Medicine, University of Pittsburgh, Pittsburgh, Pennsylvania, USA; Division of Infectious Diseases, UPMC, Pittsburgh, Pennsylvania, USA; Antibiotic Management Program, UPMC, Pittsburgh, Pennsylvania, USA

**Keywords:** candidemia, diagnostics, invasive candidiasis, stewardship, T2Candida

## Abstract

**Background:**

Diagnosis of invasive candidiasis (IC) is limited by insensitivity and slow turnaround of cultures. Our objectives were to define the performance of T2Candida, a nonculture test, under guidance of a diagnostic stewardship program, and evaluate impact on time to antifungal initiation and antifungal utilization.

**Methods:**

This was a retrospective study of adult medical intensive care unit (MICU) patients with septic shock for whom T2Candida testing was performed from March 2017 to March 2020. Patients with positive T2Candida results during this period were compared to MICU patients who did not undergo T2Candida testing but had septic shock and blood cultures positive for *Candida* from January 2016 through March 2020.

**Results:**

Overall, 155 T2Candida tests from 143 patients were included. Nine percent of T2Candida tests were positive compared to 4.5% of blood cultures. Sensitivity, specificity, positive predictive value, and negative predictive value of T2Candida for proven and probable IC were 78%, 95%, 50%, and 99%, respectively. Patients who tested positive for T2Candida (n = 14) were diagnosed earlier and initiated on antifungal therapy sooner than patients with IC (n = 14) diagnosed by blood culture alone (median, 5.6 vs 60 hours; *P* < .0001). Median antifungal days of therapy/1000 patient-days were 23.3/month preimplementation and 15/month postimplementation (*P*  *=* .007). Following a negative T2Candida result, empiric antifungals were either not administered in 58% or discontinued within 72 hours in 96% of patients.

**Conclusions:**

Diagnostic stewardship guided T2Candida testing resulted in reduced time to IC diagnosis, faster initiation of antifungal therapy, and lower antifungal usage among MICU patients with septic shock.

Invasive candidiasis (IC) includes candidemia and deep-seated candidiasis. Candidemia is associated with an attributable mortality of 20%–40% [1], which is even higher among patients with septic shock [2]. Blood cultures are the diagnostic gold standard for candidemia, yet they fail to identify 50% of IC and are limited by slow turnaround times [3]. T2Candida is a United States Food and Drug Administration–approved, direct from blood test for candidemia that amplifies *Candida* nucleic acid and detects amplicons with magnetic resonance. It detects 5 major *Candida* species (*C albicans*, *C tropicalis*, *C glabrata*, *C krusei*, *C parapsilosis*) within 5 hours [4]. A meta-analysis of mainly clinical trial data defined T2Candida pooled sensitivity of 91% and specificity of 94% [5]. In clinical practice, T2Candida demonstrates lower sensitivity (65%–73%) but comparable specificity (96%) for detection of candidemia [6, 7].

Two studies have demonstrated improved time to antifungal initiation following the implementation of T2Candida [6, 8]. The impact of T2Candida on empiric antifungal use in patients at risk for candidemia is less clear. In 1 study, average micafungin days of therapy (DOT) decreased from 6.7 days to 2.4 days following T2 implementation; however, 41.6% of patients continued to receive micafungin without mycological evidence of infection [6]. In other studies, empiric antifungal therapy was discontinued within 48 hours in fewer than half of patients with a negative T2Candida test [9, 10]. We propose that defined management algorithms with oversight by a diagnostic stewardship team are needed to realize the full impact of T2Candida on clinical outcomes and antifungal utilization. Such algorithms should define the patient populations most likely to benefit from the test, as the performance of T2Candida, like other nonculture diagnostics, is dependent upon the prevalence of disease [11].

The prevalence of candidemia increases from approximately 1% to 10% across at-risk patient populations [12]. For T2Candida to be most useful to clinicians, a positive test should be associated with high enough probability of IC to warrant antifungal treatment, while a negative test should justify discontinuing treatment [3, 11]. The evaluation of real-world data for T2Candida is hindered by its use in patient populations with varying IC risk. In this study, we implemented a standardized diagnostic stewardship algorithm for T2Candida in our medical ICU (MICU). Our main objectives were to define T2Candida performance under the guidance of diagnostic stewardship and assess the impact on time to antifungal initiation and antifungal utilization. We anticipated that patients with septic shock would have pretest likelihood of candidemia between 3%–7% and that T2Candida positive and negative predictive values (PPV and NPV, respectively) would be sufficient to direct antifungal treatment decisions [12].

## METHODS

### Study Design

This was a retrospective study of adult patients with septic shock residing in a 32-bed MICU at an academic center for whom T2Candida testing was performed between March 2017 and March 2020. MICU clinicians, pharmacists, and nurses were educated on testing criteria and appropriate sample collection prior to initiation of the study (Supplementary Appendix A). Primary teams seeking T2Candida testing were required to call diagnostic stewardship for preauthorization. Infectious diseases (ID) consultation was not mandatory to order T2Candida. The diagnostic stewardship team was comprised of ID physicians, ID fellows, ID pharmacists, and clinical microbiologists. Testing was restricted to MICU patients with septic shock requiring vasopressors for ≥3 hours (Supplementary Appendix A). Upon approval, teams were instructed to draw blood cultures with T2Candida tests. Approved tests were performed in real time from 6:00 Am to 1:30 Pm, Monday through Friday. Samples collected outside of this window were stored for testing the next morning. For inclusion in this study, T2Candida tests from the same patient were included and considered independent events if they were separated by ≥7 days. Any patient who received >72 hours of antifungal therapy (>3 doses of fluconazole or echinocandin) prior to testing or had an invalid T2Candida test result without a valid repeat test was excluded.

### Patient Consent Statement

This study was reviewed by the University of Pittsburgh Institutional Review Board and qualified for “exempt” status as defined by federal regulation (STUDY22070065). The need for informed consent was waived for this project deemed to pose minimal risk to patients.

### Definitions

Severity of acute illness was assessed by Pitt bacteremia score [13–15]. *Candida* colonization was defined as recovery of *Candida* at ≥2 sites, including respiratory tract secretions, skin, wound or urine. Culture of extrablood sites was at the discretion of the primary team. *Candida* score and Mycoses Study Group Education and Research Consortium (MSGERC) *Candida* clinical prediction rule were evaluated as previously defined [16, 17].

IC cases were categorized using modified European Organization for Research and Treatment of Cancer (EORTC)/MSGERC definitions of invasive fungal disease [18, 19]. Proven IC was defined as a positive blood culture for *Candida* within 1 day of T2Candida testing. Probable IC was defined as presence of (1) a clinical criterion (eg, compatible ocular findings by fundoscopic examination, hepatosplenic lesions by computed tomography) or mycological evidence (eg, recovery of *Candida* from a sterile site), (2) clinical or radiologic (nonpulmonary) signs of infection compatible with IC otherwise unexplained, and (3) ≥1 of the following risk factors: glucocorticoids, neutropenia, presence of central venous catheter (CVC), renal replacement therapy, total parenteral nutrition, intra-abdominal surgery, or solid organ transplant. Possible IC has not been well defined for nonimmunocompromised hosts [18]. For this study, we defined possible IC as (1) a negative blood culture but a positive T2Candida result, (2) no clear alternative etiology of septic shock, and (3) ≥1 risk factor. A false-positive T2Candida test was defined as a positive T2Candida result with negative blood cultures for *Candida* and clinical signs of infection attributable to an alternative explanation (eg, bacteremia). A false-negative T2Candida test was defined as a positive blood culture for *Candida* and a negative T2Candida result. Sensitivity and specificity were calculated using positive blood culture results for a *Candida* species as reference.

### Patient Outcomes and Antifungal Utilization

We compared patients with a positive T2Candida test during the study period to a control group. The control group consisted of consecutive MICU patients with septic shock and positive blood cultures for *Candida* who did not undergo T2Candida testing. All control patients met the inclusion and did not have factors included in the exclusion criteria described above. These control patients were selected in reverse chronological order from March 2020 to January 2016 until the sample size matched the 14 patients with positive T2Candida. The primary outcome was time to appropriate antifungal therapy from the time of T2Candida or blood culture collection. Secondary outcomes included time to detection of candidemia, time to source control, length of hospital stay, all-cause in-hospital mortality, 30-day mortality, and total antifungal utilization.

Antifungal utilization was defined as DOT per 1000 patient-days (PD) among patients in the MICU without mycologic evidence of infection before and after implementation of T2Candida. Preintervention antifungal utilization was determined for the year preceding T2 implementation (February 2016 through February 2017). The postintervention phase included the entire study period from March 2017 through March 2020.

### Statistical Analysis

Continuous variables were reported as median with interquartile range (IQR), and differences between groups were compared using the Mann-Whitney *U* test. Fisher exact test was used to assess a statistical relationship between categorical variables. *P* values <.05 (2-tailed) were considered significant.

## RESULTS

### Patient Characteristics

**Figure 1.**
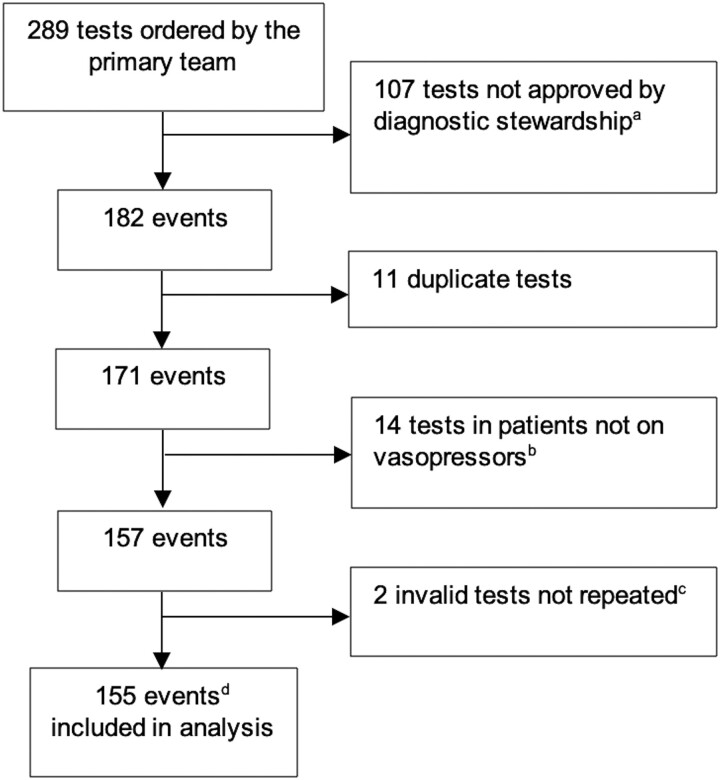
Flowchart of T2Candida testing in the medical intensive care unit during the study period. ^a^Tests were not approved by diagnostic stewardship if requests did not meet testing criteria (see Supplementary Appendix A). ^b^Tests were performed in these patients, but it was later discovered that they did not require vasopressors for ≥3 hours at time of testing and therefore results were excluded from analysis. ^c^If an invalid test was repeated and produced a valid result, the valid result was included. ^d^Includes 155 T2Candida tests from 143 patients. T2Candida tests from the same patient were considered independent, unique events if they were separated by at least 7 days.

Over the study period, 289 T2Candida tests were requested. Of the 107 T2Candida orders denied by our diagnostic stewardship team for not meeting testing criteria (Supplementary Appendix A), only 1 patient subsequently developed candidemia (Figure 1). After exclusion of an additional 27 patients (Figure 1), 155 T2Candida tests from 143 patients were included in the analysis. Demographics and clinical characteristics are summarized in Table 1. Twenty-two percent of patients had received ≥1 dose (but ≤72 hours) of fluconazole or an echinocandin immediately prior to T2Candida sample collection. The median time between blood culture and T2Candida collection was 7.5 hours (IQR, 0–19.5). Fourteen percent of patients had a *Candida* score ≥3. Only 6% and 3% of patients had pancreatitis and prior abdominal surgery, respectively. At the time of T2Candida testing, 39% of patients met the MSGERC clinical prediction rule for patients at risk for IC in the ICU [17].

**Table 1.** Characteristics of Study Population at Time of T2Candida Testing

Characteristic	No. (%) or Median (IQR)
Age, years	62 (52–69)
Male	85 (55)
Bacteremia	24 (15)
Antifungal use at time of T2Candida test	34 (22)
Antifungal use at time of blood culture	17 (11)
Time between BCx and T2Candida collection, h	7.9 (0–19.5)
Pitt Bacteremia Score	6 (4–7)

Risk Factors	No. (%)
ICU >48 h	108 (70)
History of IVDU	7 (4.5)
History of solid organ transplant	22 (14)
Mechanical ventilation	97 (63)
Broad-spectrum antibiotics	155 (100)
Abdominal surgery within 7 d	5 (3.2)
Total parenteral nutrition	9 (5.8)
Pancreatitis	9 (5.8)
Central venous catheter	155 (100)
Renal replacement therapy	
CRRT	54 (35)
HD or PD	14 (9)
Neutropenia^a^	5 (3.2)
Immunosuppression^b^	22 (14)
Glucocorticoids^c^	71 (46)
*Candida* colonization ≥1 site	34 (22)
*Candida* colonization ≥2 site	3 (1.9)
*Candida* score ≥3	22 (14)
Clinical prediction rule positive	61 (39)

Continuous and discrete variables are expressed as median (IQR) and No. (%), respectively.

Abbreviations: BCx, blood culture; CRRT, continuous renal replacement therapy; ICU, intensive care unit; IQR, interquartile range; IVDU, intravenous drug use; HD, hemodialysis; PD, peritoneal dialysis.

^a^Absolute neutrophil count ≤500 cells/μL.

^b^More than 1 dose of an immunosuppressive agent (eg, tacrolimus, mycophenolate, cyclosporine).

^c^More than 1 dose of prednisone equivalent ≥20 mg/day within 7 days of testing.

### T2Candida Diagnostic Performance

Among 155 T2Candida tests, results were concordant with blood cultures in 144 (93%): 5 samples had positive T2Candida/blood culture results, and 139 had negative T2Candida/blood culture. Discordant T2Candida and blood culture results were identified in 7% (11/155) of samples, including 2 samples with T2Candida-negative/blood culture–positive results, and 9 samples with T2Candida-positive/blood culture–negative results. Overall, 9% (14/155) of samples had positive T2Candida and 4.5% (7/155) had *Candida-*positive blood culture; 10% (16/155) of samples had either a positive T2Candida or blood culture.

Characteristics of patients with positive T2Candida and/or blood culture (n = 16) are shown in Table 2. Of the 14 T2Candida-positive results, 64% (9/14) were classified as proven (n = 5), probable (n = 2), or possible (n = 2) IC. Targets identified were *C parapsilosis* and *C albicans/tropicalis* (n = 7 each) and *C glabrata/krusei* (n = 2). Among proven IC, targets detected by T2Candida matched *Candida* species identified by blood cultures in all 5 cases. Of the 2 patients with probable IC, 1 had ocular findings consistent with *Candida* endophthalmitis and the other had a positive CVC tip culture for *C parapsilosis*. Of the 2 patients with possible IC, no other sources of infection or shock were found. One patient with possible IC was treated with fluconazole and survived, and the other was treated with caspofungin but died 4 days later. Thirty-six percent (5/14) of positive T2Candida results were classified as false positives based on presence of an alternative source of infection; 4 of 5 false-positive T2Candida were due to *C parapsilosis*. Two patients had false-negative T2Candida tests. Performance characteristics of T2Candida for detecting proven and probable IC are shown in Table 3.

**Table 2.** Characteristics of Patients With Proven, Probable, or Possible Invasive Candidiasis

Age, Sex	Risk Factors^a^	T2Candida Result	BCx Result	Classification	Likely Etiology of Septic Shock	PBS/CPR (+)	Clinical Outcome
Proven IC (candidemia)							
43, M	IVDU, MV	*C parapsilosis*	*C parapsilosis*	True positive	*Candida* IE	4, no	Treated with FLC × 42 d and survived
58, F	MV, RRT, glucocorticoids	*C glabrata/ krusei*	*C glabrata*	True positive	BSI	8, yes	Died on day 2
50, F	TPN, glucocorticoids	*C glabrata/ krusei*	*C glabrata*	True positive	BSI	2, no	Treated with CAS × 14 d and survived
63, M	MV, RRT, glucocorticoids	*C albicans/ tropicalis*	*C albicans*	True positive	BSI	10, yes	Died on day 2
74, F	MV, RRT, glucocorticoids	*C albicans/ tropicalis*	*C albicans*	True positive	BSI	10, yes	Died on day 2
28, F	IVDU	Negative	*C albicans*	False negative	*Candida* IE	3, no	Treated with FLC × 42 d and survived
65, F	MV, glucocorticoids	Negative	*C albicans*	False negative	BSI	7, yes	Treated with FLC × 14 d and survived
Probable IC							
63, F	RRT, glucocorticoids	*C parapsilosis*	Negative	Probable IC^b^	BSI	5, no	Treated with FLC × 14 d and survived
69, F	MV, RRT	*C albicans/ tropicalis*	Negative	Probable IC^c^	BSI	6, yes	Died on day 6
Possible IC							
73, M	MV, RRT, neutropenia, SOT, IS, glucocorticoids	*C parapsilosis*	Negative	Possible IC	BSI	5, yes	Treated with FLC × 14 d and survived
73, F	MV, RRT, SOT, IS, glucocorticoids	*C albicans/ tropicalis*	Negative	Possible IC	BSI	6, yes	Died on day 4
No IC							
74, F	None	*C parapsilosis*	Negative	False positive	Left hip PJI	3, no	Died on day 5
71, F	MV	*C parapsilosis*	Negative	False positive	VAP	7, no	Died on day 3
54, F	MV	*C parapsilosis*	Negative	False positive	VAP	7, no	Treated with CAS × 2 d and survived
48, M	MV, glucocorticoids	*C parapsilosis*	Negative	False positive	*Escherichia coli* bacteremia	10, yes	Died on day 4
54, F	MV, RRT, SOT, IS, glucocorticoids	*C albicans/ tropicalis*	Negative	False positive	VAP	10, yes	Died on day 3

Abbreviations: BCx, blood culture; BSI, bloodstream infection; CAS, caspofungin; CPR, clinical prediction rule; F, female; FLC, fluconazole; IC, invasive candidiasis; IE, infective endocarditis; IS, immunosuppression; IVDU, intravenous drug use; M, male; MV, mechanical ventilation; PBS, Pitt Bacteremia Score; PJI, prosthetic joint infection; RRT, renal replacement therapy; SOT, solid organ transplant; TPN, total parenteral nutrition; VAP, ventilator-associated pneumonia.

^a^All patients had central venous catheter (CVC) and broad-spectrum antibiotics.

^b^CVC culture positive for *C parapsilosis*.

^c^Endophthalmitis right eye.

**Table 3.** Diagnostic Performance of T2Candida for Proven and Probable IC^a^

Performance Characteristics	Proven IC	Proven and Probable IC
Sensitivity, %	71.4	77.8
Specificity, %	93.9	95.2
LR^+^	11.8	16.2
LR^−^	0.3	0.23
Prevalence, %	4.5	5.8
PPV, %	35.7	50
NPV, %	98.6	98.6

Abbreviations: LR^+^, positive likelihood ratio; LR^–^, negative likelihood ratio; NPV, negative predictive value; PPV, positive predictive value.

^a^Assumes probable IC cases to be true positives that were missed by blood culture.

### Antifungal Treatment Outcomes

To evaluate the impact of stewardship-guided T2Candida on antifungal treatment outcomes, we compared 14 patients who had positive T2Candida with 14 consecutive MICU patients with septic shock and blood cultures positive for *Candida* who did not undergo T2Candida testing (Table 4). The clinical characteristics of the patients are presented in Supplementary Table 1. The T2Candida cohort had a numerically higher proportion of glucocorticoid use. *Candida parapsilosis* was detected more frequently in the T2Candida group compared to the blood culture group (50% vs 0%; *P*  *=* .006). Two patients in the blood culture group had *Candida* spp (*C dubliniensis* and *C lusitaniae*) that would not have been detected by T2Candida.

**Table 4.** Outcomes of Patients With Positive T2Candida Versus Blood Cultures

Outcome	T2Candida Positive (n = 14)	BCx Positive (n = 14)	*P*-value
Time to detection of candidemia, h	16.7 (7.5–19)	56 (34–80)	<.0001
Time to antifungal therapy, h	5.6 (−5.8 to 8)^a^	60 (41–84)	<.0001
Source control procedure^b^	7 (50)	11 (79)	.237
Time to source control, h	9 (−2 to 40)	76 (11–106)	.151
Length of hospital stay, d	15.5 (8–29)	16 (10–39)	.659
In-hospital mortality	9 (64)	6 (43)	.450
30-d mortality	10 (71)	6 (43)	.252

Continuous and discrete variables are expressed as median (interquartile range) and No. (%), respectively.

Abbreviation: BCx, blood culture.

^a^Four patients in the T2Candida-positive cohort were started on antifungals prior to T2Candida collection due to the test being requested after operating hours; all 4 received <14 hours of antifungal before invasive candidiasis diagnosis. Time to antifungal treatment was negative in these patients.

^b^Source control was defined as removal of any preexisting central venous catheter or documented surgical or radiologic procedure to drain fluid collections thought to be the source of *Candida* infection.

Time to initiation of antifungal therapy was shorter in the T2Candida group compared to the blood culture group (median, 5.6 vs 60 hours; *P* < .0001). Patients in the T2Candida group trended toward shorter median time to source control than the blood culture group (9 vs 76 hours, respectively; *P* = .237). Hospital lengths of stay and the 30-day mortality rate were not significantly different between groups.

### Antifungal Utilization

**Figure 2.**
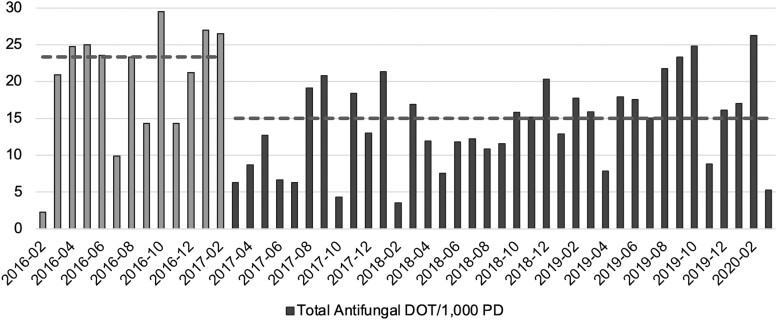
Antifungal days of therapy (DOT) per 1000 tpatient-days (PD) before and after T2Candida implementation in the medical intensive care unit (MICU). DOT was calculated monthly and standardized by patient days in the MICU. Dashed lines represent median antifungal DOT/1000 PD for the specified preimplementation and postimplementation period. Median DOT/1000 PD was 23.3 days/month preimplementation and 15 days/month postimplementation (*P* = .007).

Median antifungal DOT/1000 PD among MICU patients without mycological evidence for IC in the preimplementation period was 23.3 days/month compared to 15 days per month in the postimplementation period (*P*  *=* .007; Figure 2). Fifty-seven percent of patients with negative T2Candida did not receive empiric antifungal therapy. Within 48 and 72 hours, 84.9% and 95.7% of patients, respectively, either did not receive antifungals or had their antifungal discontinued. The number of T2Candida tests needed to avoid a 3-day course of antifungal treatment was 2.6.

**Figure 3.**
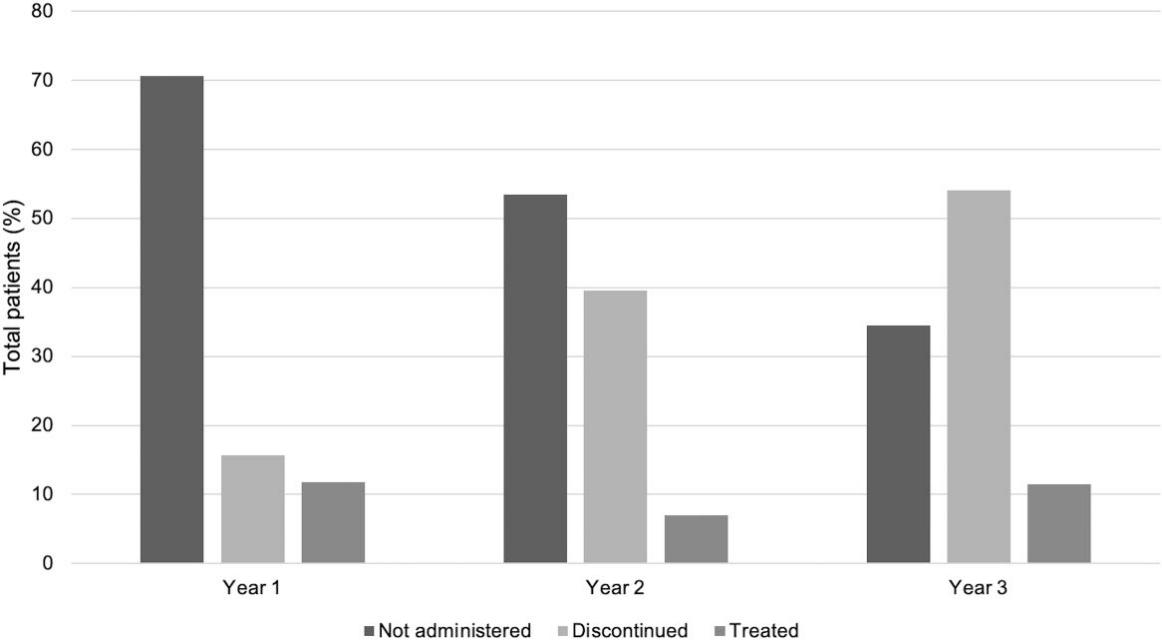
Antifungal use trends following T2Candida by year. Following a negative T2Candida result, antifungals were either not administered or discontinued within 48 and 72 hours in 84.9% and 95.7% of cases, respectively. We observed a gradual decrease in the proportion of patients who did not receive any antifungals from 70.6% in the first year to 34.4% in the third year. These data corresponded to an increase in empiric antifungal use from 15.7% to 54.1% over the same period (*P* < .001).

When antifungal use was analyzed by postimplementation year (Figure 3), the rate of patients who did not receive any antifungals gradually decreased from 70.6% in the first year to 34.4% in the third year; these data corresponded to an increase in empiric antifungal use from 15.7% to 54.1% (*P* < .001). The rate of patients remaining on antifungals 72 hours after the T2Candida test remained stable throughout the 3 years. The 30-day mortality rates did not vary over the study period.

## DISCUSSION

T2Candida testing guided by diagnostic stewardship among MICU patients with septic shock resulted in a faster time to diagnosis and treatment of IC while decreasing overall antifungal utilization. In our study population, the prevalence of IC was 4.5% when identified by blood culture alone and 7.1% when blood cultures were combined with T2Candida. These data are well-aligned with our a priori predicted prevalence of 3%–7% [11, 12]. By employing strict testing criteria that required stewardship approval, we evaluated a patient cohort in which the disease prevalence was sufficiently high to warrant diagnostic testing but not high enough to warrant antifungal prophylaxis [11]. Overall, the T2Candida PPV was 50% among patients with proven and probable IC, and NPV was 98.6%, thresholds at which antifungal initiation and restriction could be justified, respectively. Furthermore, our stewardship team denied T2Candida testing in 107 patients, among whom prevalence of IC was <1% (1/107). In contrast to T2Candida performance, sensitivity and PPV of the MSGERC *Candida* prediction rule in MICU patients with septic shock were only 57% (4/7) and 7% (4/61), respectively. Therefore, targeted use of T2Candida may be more effective for antifungal stewardship than risk stratification based on a constellation of clinical parameters among patients with septic shock. Our experience can serve as a model for centers and stewardship programs interested in incorporating T2Candida or other nonculture diagnostics into rational patient management strategies.

Overall, sensitivity (78%) and specificity (95%) of T2Candida for proven and probable IC was comparable to rates previously reported of 65%–73% and 96%, respectively [6, 7]. Discordant T2Candida and blood culture results were observed in 7% of tests. Negative blood culture/positive T2Candida was observed in 6% of tests (9/155); 44% (4/9) of these results were associated with probable or possible IC, and 56% (5/9) were considered false positives. Such findings might be explained by nonviable *Candida* in the bloodstream, transient translocation of *Candida* nucleic acid due to intestinal barrier dysfunction in septic shock [20], or sample contamination [21]. Eighty percent of false-positive cases were attributed to *C parapsilosis*, a skin commensal [22]. Our findings emphasize the importance of aseptic technique before sampling and discourage T2Candida blood draws via indwelling intravascular catheters.

One percent (2/155) of cases showed positive blood culture but negative T2Candida, and both cases were associated with *C albicans* fungemia. These false-negative T2Candida results may be explained by time between sample collection or lack of DNA detection. The median time between T2Candida sample collection and blood cultures was 7.9 hours but ranged significantly across patients. Data show that up to 75% of candidemia is transient, and subsequent blood cultures might be negative even in the absence of antifungal therapy [4, 23, 24]. Our findings demonstrate an important caveat to studying T2Candida in real-world settings where, even with diagnostic stewardship guidance, samples are not reliably collected simultaneously. Alternatively, circulating DNA during candidemia might be below the limits of detection for T2Candida, which is reported to be 1 colony-forming unit/mL of whole blood [25]. Last, the *C albicans* causing candidemia in these patients might have variability in DNA targets that might affect the binding of polymerase chain reaction primers.

Consistent with prior reports [6, 8], we identified a significant reduction in the time to initiation of antifungal therapy among patients with candidemia detected by T2Candida (median, 5.6 hours) compared to those detected by blood cultures alone (median, 60 hours). Our institutional approach included direct reporting of T2Candida results to the diagnostic stewardship team, who then communicated real-time results and antifungal recommendations to the MICU team. Such interactions cannot be understated, as several studies have shown that rapid diagnostic tests lead to more impactful outcomes when they are facilitated by antibiotic stewardship program (ASP) [26]. Our diagnostic stewardship team took advantages of these interactions to educate clinicians on the value and limitations of T2Candida testing and opportunities for prompt source control (eg, CVC removal). Indeed, we noted a trend toward faster time to source control among patients with candidemia identified by T2Candida.

Despite more rapid time to antifungal therapy and source control in the T2Candida cohort, we were not able to show survival benefit or shortened time to discharge. Several factors likely contributed to these findings. First, our small sample size limited our analytic and statistical capabilities. Second, biases may affect physicians’ requests for T2Candida testing (eg, physicians might request more tests in patients with septic shock who are rapidly deteriorating). Third, it is possible that, with an overall mortality rate of 64%, it is difficult to distinguish attributable mortality from death caused by underlying diseases [27].

In this study, T2Candida testing in conjunction with diagnostic stewardship led to a significant monthly decrease of 8.3 days in antifungal DOT/1000 PD compared to the preintervention utilization data. Indeed, antifungal avoidance and rapid discontinuation of empiric therapy were achieved in 84.9% and 95.7% of patients within 48 and 72 hours, respectively. Comparatively, a prior retrospective study of 628 T2Candida tests showed that antifungals were held or discontinued within 48 hours in 78.9% of patients; however, patients who continued on antifungal therapy beyond 48 hours had a mean DOT of 7.8 days [9]. In a more recent retrospective study, a negative T2Candida result in absence of diagnostic stewardship support prompted cessation of antifungal therapy in only 33% of patients [10]. Using our algorithm, 2.6 [1 / [95.7% – 57.6%]) T2Candida tests were needed to avoid a 3-day course of antifungals.

The optimal use of T2Candida to guide antifungal therapy remains uncertain. There are 2 possible approaches. In the first, antifungal therapy can be initiated and then discontinued rapidly following a negative result. Alternatively, positive results can guide initiation of antifungal therapy. The benefits of a “treat and stop” approach include early treatment initiation for patients with candidemia at the expense of an aggregate increase in antifungal utilization. This treatment strategy is evident in the T2Candida-positive group, where 8 of 14 patients were started on antifungal therapy prior to the T2Candida result. A “test and treat” approach would likely lead to decreased antifungal usage but may delay timely antifungal therapy for patients with septic shock. Of note, clinicians at our center transitioned over time from a “test and treat” (70.6% and 34.4% in the first and third years, respectively) to a “treat and stop” approach (Figure 3). The reason for this change in approach is unclear; however, we speculate that T2Candida implementation raised awareness of IC, and, with experience, clinicians gained confidence in the test's NPV.

This study was limited by its single-center, retrospective design. However, it lays the groundwork for future prospective studies and broader implementation of T2Candida using locally informed, evidence-based testing algorithms. Our diagnostic stewardship team approved and interpreted every T2Candida test result; therefore, any clinical benefits derived solely from T2Candida testing cannot be calculated without dedicated stewardship resources. We acknowledge that not all patients with septic shock had T2Candida testing during the study period. Our protocol was dependent upon MICU teams identifying patients for testing, and T2Candida testing was only offered during the daytime, not including weekends. Additionally, we did not reliably collect invalid T2Candida rate data during our study period; however, an internal review revealed that our institutional T2Candida invalid rate across all study locations was 8.2%, which is consistent with literature reports [7, 28]. Finally, our study focused on patients in the MICU with septic shock, and results cannot necessarily be extrapolated to other patient populations with a higher prevalence of IC or those less likely to have IC, such as those in surgical or trauma ICUs following abdominal surgery [29].

In conclusion, T2Candida testing governed by diagnostic stewardship in a well-defined patient population validated the diagnostic performance and allowed for a tailored antifungal management pathway. Our results demonstrated that T2Candida testing in conjunction with ASP intervention improved the time to antifungal initiation among patients with IC, and negative test results were helpful in avoiding or discontinuing antifungal therapy, resulting in a significant reduction in antifungal use at our center. Further research involving broader patient populations is needed to confirm these findings, and larger studies are required to determine the impact of T2Candida testing on patient outcomes.

## Supplementary Material

ofad538_Supplementary_Data

## Supplementary Data

Supplementary materials are available at *Open Forum Infectious Diseases* online. Consisting of data provided by the authors to benefit the reader, the posted materials are not copyedited and are the sole responsibility of the authors, so questions or comments should be addressed to the corresponding author.

## Notes

***Potential conflicts of interest.*** All authors: No reported conflicts.
