# Supplementary material for: Stewardship-Guided T2Candida Testing Shortens Time to Antifungal Treatment and Reduces Antifungal Usage Among Medical Intensive Care Unit Patients With Septic Shock
Source: Open Forum Infect Dis. 2023 Nov 8;10(11):ofad538. doi: 10.1093/ofid/ofad538 (PMC10651185; doi:10.1093/ofid/ofad538)
Supplement: ofad538_Supplementary_Data [file ofad538_supplementary_data.docx]

**Appendix A. T2Candida testing criteria and antifungal management for medical ICU patients in septic shock**

| **T2Candida ordering criteria:**  MICU patient with suspected infection requiring vasopressors |
| --- |
| **Patients identified by MICU primary team**  Requests approval of T2Candida from diagnostic stewardship |
| **Order T2Candida^1^**  Ordered with at least one set of peripheral blood cultures |
| **Whole blood sample sent to Microbiology**  Samples run in real-time from 06:00 to 13:30 (Mon – Fri)  Results reported to diagnostic stewardship and recorded in EMR |
| **Diagnostic stewardship intervention**  Contact primary team with result, interpret findings and provide recommendations |
| **T2Candida Result (Day 1)** |


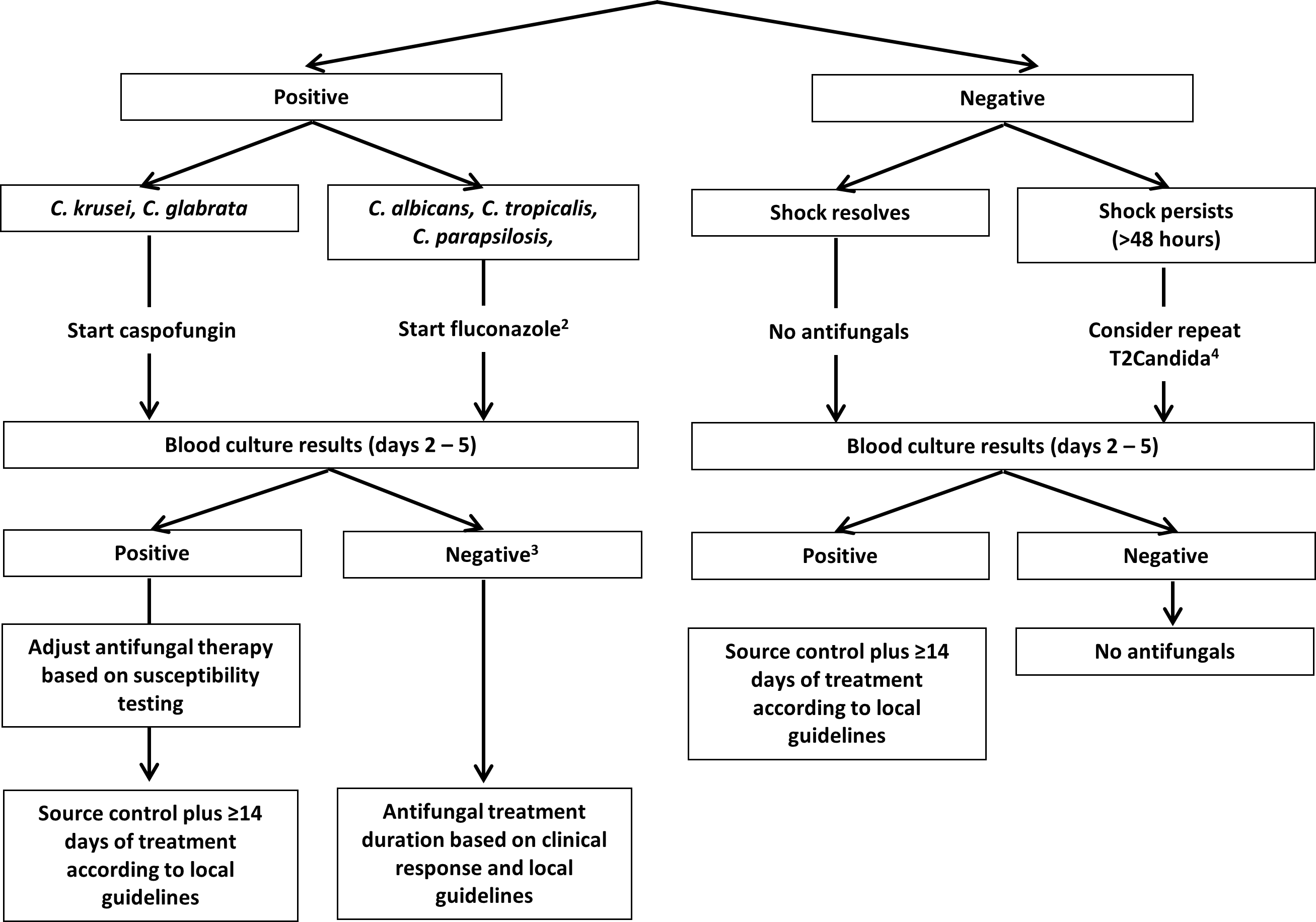


Abbreviations: MICU, medical intensive care unit; EMR, electronic medical record.

1. The diagnostic stewardship team comprised infectious disease physicians, ID fellows, ID pharmacists, and clinical microbiologists

2. One 4mL whole blood sample was collected in a purple top EDTA tube. Tests ordered during the daytime were approved by the stewardship team, overnight orders were approved by stewardship team prior to the morning batch run.

3. All antifungals, except for fluconazole, require prior authorization from our antimicrobial stewardship team. Caspofungin was recommended as an alternative for patients with a history of prior azole use, which was defined as ≥ 7 days of therapy within the past 60 days.

4. The significance of T2 (+) tests in the setting of negative blood cultures is unknown. During validation studies, false-positive results were encountered. Thus, we recommend discontinuing antifungal therapy after a short course of therapy (< 7 days) if patient exhibited an appropriate clinical response (resolution of fevers, leukocytosis, hemodynamic instability) given that short-course antifungal therapy has been shown to be effective among patients with uncomplicated candidemia. For extended treatment, we recommended Infectious Disease consult for further recommendations.

5. T2Candida could be reordered only once per week for patients in refractory shock. In general, patients with a positive T2Candida result did not undergo repeat confirmatory testing.

| **Supplementary Table 1: Characteristics of patients with positive T2Candida vs BCx** | | | |
| --- | --- | --- | --- |
|  | **T2Candida positive (n=14)** | **BCx positive (n=14)** | ***P*-value** |
| Age, years | 63 (54 – 72) | 53 (44 – 66) | 0.159 |
| Men | 4 (30) | 6 (40) | 0.695 |
| Antifungal at time of test collection | 4 (29) | 1 (7.4) | 0.326 |
| Transthoracic echocardiogram | 6 (43) | 11 (79) | 0.120 |
| *Candida* Infective Endocarditis | 1 (7.1) | 1 (7.1) | 0.999 |
| Ophthalmology examination | 9 (64) | 10 (71) | 0.999 |
| Ocular candidiasis | 1/9 (11) | 2/10 (20) | 0.999 |
| Infectious disease consult | 12 (86) | 12 (86) | 0.999 |
| Pitt Bacteremia Score | 6.5 (5 - 9.5) | 6 (4 - 7) | 0.639 |
| ***Microbiology*** | | | |
| *Candida* species identified |  |  |  |
| *C. albicans/tropicalis* | 5 (36) | 4 (28.6) | 0.999 |
| *C. glabrata/krusei* | 2 (14.3) | 7 (50) | 0.103 |
| *C. parapsilosis* | 7 (50) | 0 (0) | **0.006** |
| other *Candida* species | 0 (0) | 2 (14.3) | 0.482 |
| multiple *Candida* species | 0 (0) | 1 (7.1) | 0.999 |
| ***Risk Factors*** | | | |
| ICU > 48 hours | 11 (79) | 8 (57) | 0.420 |
| History of IVDU | 1 (7.1) | 2 (14.3) | 0.999 |
| History of solid organ transplant | 3 (21.4) | 3 (21.4) | 0.999 |
| Mechanical ventilation | 11 (79) | 9 (64) | 0.678 |
| Broad spectrum antibiotics | 14 (100) | 14 (100) | 0.999 |
| Abdominal surgery | 0 (0) | 0 (0) | 0.999 |
| Total parenteral nutrition | 1 (7.1) | 1 (7.1) | 0.999 |
| Pancreatitis | 0 (0) | 0 (0) | 0.999 |
| Central venous catheter | 14 (100) | 14 (100) | 0.999 |
| Renal replacement therapy | 8 (57) | 8 (57) | 0.999 |
| Neutropenia | 1 (7.1) | 1 (7.1) | 0.999 |
| Immunosuppression | 3 (21.4) | 2 (14.3) | 0.999 |
| Glucocorticoids | 9 (64) | 5 (36) | 0.257 |
| *Candida* colonization ≥ 1 site | 8 (57) | 7 (50) | 0.999 |
| *Candida* score ≥ 3 | 1 (7.1) | 1 (7.1) | 0.999 |
| Clinical prediction rule positive | 8 (57) | 7 (50) | 0.999 |
| Abbreviations: BCx, Blood culture; IVDU, Intravenous drug use.  Continuous and discrete variables are expressed as median (IQR) and n (%), respectively | | | |
